# Aircraft Fuselage Corrosion Detection Using Artificial Intelligence

**DOI:** 10.3390/s21124026

**Published:** 2021-06-11

**Authors:** Bruno Brandoli, André R. de Geus, Jefferson R. Souza, Gabriel Spadon, Amilcar Soares, Jose F. Rodrigues, Jerzy Komorowski, Stan Matwin

**Affiliations:** 1 Department of Computer Science, Institute for Big Data Analytics, Dalhousie University, Halifax, NS B3H 1W5, Canada; 2 Department of Computer Science, Federal University of Uberlandia, Uberlandia 38400-902, Brazil; geus.andre@ufu.br (A.R.d.G.); jrsouza@ufu.br (J.R.S.); 3 Institute of Mathematics and Computer Science, University of Sao Paulo, Sao Carlos 13566-590, Brazil; gabriel@spadon.com.br (G.S.); junio@icmc.usp.br (J.F.R.J.); 4 Department of Computer Science, Memorial University of Newfoundland, St. John’s, NL A1C 5S7, Canada; amilcarsj@mun.ca; 5 JPWK Aerospace, Ontario, ON K1A 0R6, Canada; jerzy.komorowski@JPWKaero.com; 6 Institute for Computer Science, Polish Academy of Sciences, 01-248 Warsaw, Poland

**Keywords:** aircraft corrosion inspection, automatic corrosion detection, material fatigue, corrosion science, rust detection, aviation maintenance, deep learning

## Abstract

Corrosion identification and repair is a vital task in aircraft maintenance to ensure continued structural integrity. Regarding fuselage lap joints, typically, visual inspections are followed by non-destructive methodologies, which are time-consuming. The visual inspection of large areas suffers not only from subjectivity but also from the variable probability of corrosion detection, which is aggravated by the multiple layers used in fuselage construction. In this paper, we propose a methodology for automatic image-based corrosion detection of aircraft structures using deep neural networks. For machine learning, we use a dataset that consists of D-Sight Aircraft Inspection System (DAIS) images from different lap joints of Boeing and Airbus aircrafts. We also employ transfer learning to overcome the shortage of aircraft corrosion images. With precision of over 93%, we demonstrate that our approach detects corrosion with a precision comparable to that of trained operators, aiding to reduce the uncertainties related to operator fatigue or inadequate training. Our results indicate that our methodology can support specialists and engineers in corrosion monitoring in the aerospace industry, potentially contributing to the automation of condition-based maintenance protocols.

## 1. Introduction

Corrosion has been widely investigated in many areas involving materials science, including maritime vessels [1], steel pipelines [2], and concrete and steel damage [3]. Recent studies estimated that the total cost of corrosion maintenance worldwide amounts to roughly 2.5 trillion US dollars per year [4,5]. In particular, aircraft corrosion occurs throughout individual aircraft and aircraft fleets operated in diverse service environments, including temperature and humidity, compounds occurring in the atmosphere, aircraft washing practices, and icing fluids [6,7]. Corrosion is mitigated by the choices of materials, construction details, surface protection systems, and maintenance practices. If not mitigated, corrosion can undermine the structural integrity and, in some cases, contribute to fuselage fatigue [8,9]. Therefore, appropriate corrosion monitoring can significantly improve safety and the economic risks in the aircraft industry.

Typically, specialists and engineers are responsible for inspecting the aircraft structure by (i) direct or (ii) indirect methods. Direct inspection involves detecting a change in structures primarily by visual examination, usually using a flashlight to detect cracks, corrosion products, and changes related to surface deformations. Although it is considered a fast and straightforward methodology, this kind of visual inspection is subjective. It does not record any data related to the inspection or the condition of the aircraft.

In contrast, indirect detection requires the adoption of specific devices to measure material deterioration using different nondestructive technologies (e.g., eddy current, infrared, and ultrasonic thickness testing) [10,11]. The main advantage of indirect detection nondestructive testing (NDT) is a higher probability of detection and quantification of corrosion. However, because these techniques require specialized equipment that calls for highly trained operators and are time-consuming, they cannot be economically deployed to inspect the entire aircraft.

Additionally, although aircraft corrosion has been investigated for many years, several points remain under discussion. It is important to highlight that many authors have proposed different methods of sensing aircraft parts and structures, including ultrasonic sensors [12], X-ray tomography [6], lamb wave tomography (LWT) [13,14], and compressed sensing (CS) [15]. Recently, Hossein et al. [16] reviewed the state of the art of the field of nondestructive testing methods to evaluate the integrity of critical aerospace composites.

Several studies proposed the automatic detection of cracks and corrosion from images based on mathematical and computational models to overcome the drawbacks of visual inspection methodologies. The pioneering research of Prabhu and Winfree [17] for NASA used thermal sensors to collect images and neural networks for inspecting aircraft structures. Bellinger and Komorowski [18] presented studies to determine the effect of corrosion pillowing on the structural integrity of fuselage lap joints. Tan et al. [19] used a computational approach based on a fuzzy system to predict the corrosion on an aircraft.

Liao et al. investigated the effects of exfoliation corrosion on aircraft fuselages [8,12]. They developed a 3D finite element model to evaluate the effects of prior exfoliation corrosion on the residual fatigue life of wing skins (sheet metal). In 2012, Hoeppner and Arriscorreta published a review addressing studies about exfoliation and pitting corrosion [20]. An article proposed by Gialanella [21] investigated the corrosion of aluminum, magnesium, and other alloys used in aircraft structures exposed to standard service environmental conditions and high temperatures. Gialanella concluded that changes occur in the structural and aerodynamic properties of turbines. They used finite element models to analyze the rivet stress and the corrosion pillowing. Similarly, long-term evaluation was performed for the corrosion and fatigue crack analysis of military aircrafts [22]. Ganther et al. [23] investigated the influence of climate data for monitoring aircraft corrosion.

Recently, Li et al. [24] published a review of corrosion in aircraft structures for aviation monitoring. They collected data according to the flight path, time of the day, and month of the year. Although similar to the scope of our investigation, few papers applied Aritificial Intelligence, particularly Deep Learning, to the material corrosion inspection.

In recent years, Deep Neural Networks (DNNs) have witnessed striking advances in different areas with machines regarding understanding and manipulating data, including audio, time series, and image classification [25,26,27]. Although DNNs can be used to perform classification directly over aircraft surfaces, this does not capture images from the internal coating of the aircraft.

In the work of Malekzadeh et al. [28], the authors proposed an automatic defect detection over aircraft fuselage using Convolutional Neural Networks (CNNs), such as AlexNet [29] and VGG [30]. They collected external images of different airplane fuselage while small saliencies were detected using the SURF algorithm.

Similarly, Miranda et al. [31] used machine learning algorithms—SVM and CNNs—for classifying images of the exterior surface of aircraft fuselages acquired by an unmanned aerial vehicle (UAV). Different from previous studies that focused only on external surface analyses, we investigated the effects of aircraft corrosion over internal (faying) fuselage lap joints using the D-Sight non-destructive method. In this line of investigation, our methodology aimed to identify corrosion pillowing, which can impact the structural integrity of aircraft fuselage lap joints.

The main contributions of this study are listed below:

- an evaluation of the state-of-the-art Deep Learning models for corrosion monitoring;
- a Deep Learning model that can be trained using a small number of corrosion images, allowing for an automated corrosion analysis based on a reduced number of data samples labeled by specialists;
- a Deep Learning model that performs aircraft corrosion identification with startling accuracy; and
- a robust model validation achieved with experimental data and specialist supervision using a portable non-destructive device.

## 2. Materials and Methods

### 2.1. Aircraft Fuselage

In this work, we consider transport aircraft fuselages that have been assembled from 2024-T3 alloy skins with 7075-T6 stringers and frames. The longitudinal joints are either lap or butt splices, while the circumferential joints are usually butt joints. These joints were riveted; in some cases, they were additionally bonded. A typical joint consists of two or more sheet material layers with a stringer, tear-strap, or frame attached on the inside. To prevent or delay the onset of faying surface corrosion, sealants are also used.

**Figure 1:**
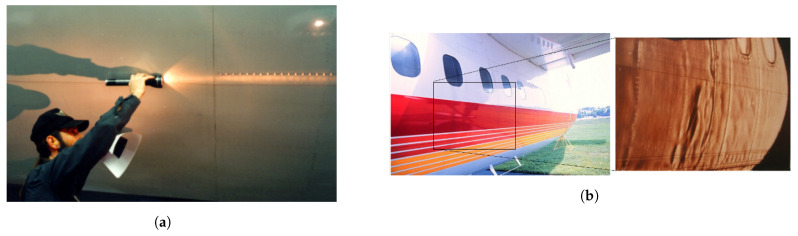
An example showing pillowing corrosion and surface micro deformations. (**a**) A specialist using a flashlight performs fuselage visual inspection; (**b**) the fuselage photo scanned showing surface micro perturbations caused by corrosion.

Since the interior and exterior of the fuselage may corrode, it is far more relevant and challenging to identify the corrosion of joint faying surfaces. Typically in the early stages, corrosion is in the form of filiform or pitting corrosion. Figure 1 shows an example of pillowing surface micro perturbations assessed visually by an expert. This is a significant issue for joint faying surfaces that are not easily accessible. Faying surface corrosion occurs between the riveted sheets or between the sheets and formers in fuselage joints. This type of corrosion is usually associated with small volumes of stagnant solution, which become trapped and, depending on the chemical nature, produces a combination of pitting, galvanic, and exfoliation corrosion.

**Figure 2:**
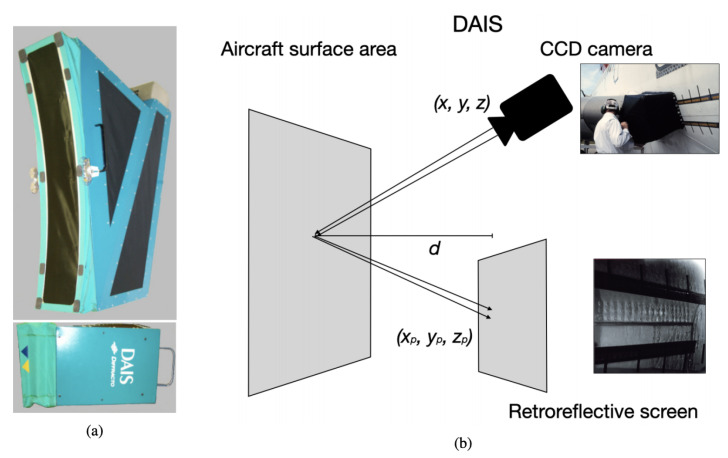
D-Sight Aircraft Inspection System (DAIS). (**a**) DAIS 250c, and (**b**) schematic of DAIS functioning.

**Figure 3:**
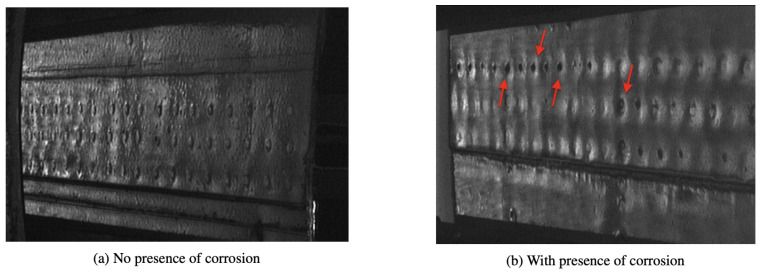
Two samples taken with the DAIS 250c device of a simple shear lap joint. (**a**) An image with no corrosion; and (**b**) the corrosion pillowing affecting rivets in red color.

Different corrosion products are relevant to this investigation; such products contain a mix of oxides, primarily aluminum oxide trihydrate. This is significant because the molecular volume ratio of this oxide to the alloy from which it originated is very high. Since these corrosion products are insoluble, they tend to remain within the joint. Furthermore, the high molecular volume ratio of the corrosion product is responsible for the deformation of the skins in a joint, which causes the appearance of corroded fuselage joints, commonly referred to as pillowing (see Figures 2b and 3).

### 2.2. D-Sight Aircraft Inspection System (DAIS)

D-Sight Aircraft Inspection System (DAIS) is a portable non-destructive device for visual analysis of aircraft surface areas aimed at inspecting fuselage joints. This system contains a CCD camera and a double pass retroreflection projected onto a screen. Figure 2 illustrates a conceptual workflow of DAIS for supporting the aircraft inspection.

The first model of DAIS was developed in the early 1990s by the Institute for Aerospace Research of the National Research Council Canada (IAR/NRCC) and Diffracto Limited in conjunction with the Transportation Development Centre of Transport Canada, and the Technical Center of the U.S Federal Aviation Administration. IAR/NRCC collaborated with this project, as explained in Section 3.1. DAIS was created aiming at supporting the surface inspection of riveted aircraft joints only.

After several improvements, the DAIS 250C was released in the market, as illustrated in Figure 2a. The device uses a simple optical arrangement and is capable of detecting changes in surface topography greater than 5 μm. Details of the optical DAIS arrangement are observed in Figure 2b, which contains the CCD projected over a plan with an array of sensors where images are electronically collected. The equipment is placed over curved aircraft surfaces, and images are generated while the inspection is carried out.

### 2.3. AI and Deep Neural Networks

Over the last decade, Artificial Intelligence (AI) has revolutionized many areas, e.g., medicine, e-banking, autonomous cars, ecology, and agriculture [32,33,34,35]. Particularly, Deep Neural Networks (DNNs) have shown the ability to achieve or even surpass human-level performance in many visual tasks [36,37]. A class of these DNNs are so-called Convolutional Neural Network (shortly CNNs), and these have had a tremendous impact on several fields in science. They have lead to significant improvements in speech recognition, image recognition, and autonomous agents. The majority of such tasks were considered to be impossible to be solved by computers before the advent of deep learning.

A significant advance towards deep neural networks is the use of specialized layers, including convolution and pooling layers, to enable the model locality of parameters and dramatically reduce the scale of the input during the model training or feature learning [38]. The main advantage of the convolution layers is that such operation considers a local neighborhood of neurons rather than all neurons of the same layer, while pooling is responsible for reducing the scale of the input for the next layers. Several proposed deep learning architectures [39,40] use multiple blocks of successive convolution and pooling operations for many tasks.

By learning shared feature representations, the network training uses backpropagation based on gradient descent associated with a loss function that measures the quality of the weight set. Afterward, we can represent a single-layer fully connected network with non-linear activations simply as $\hat{y}=\left(\hat{f}\right)=Wx$ calculated as matrix multiplication. By means of an L2-loss, we end up with the following:

(1) $$L\left(\theta \right)=\left|\right|\left(\hat{f}\left(\left(x\right)\right)\right)-{y||}_{2}^{2}=\frac{1}{2}||Wx-{y||}_{2}^{2}$$

In order to update the parameters θ=W, we basically compute the partial derivatives $\frac{\partial L}{\partial W}$ using the chain rule. Then, the final weight update is calculated as:

(2) $$W^{i+1}=W^{i}+\eta \;\left(W^{i}x-y\right)x^{T}$$

where η is the so-called learning rate and *i* is the number of epochs. Let us assume a network structure with four layers:

(3) $$L\left(\theta \right)=\hat{f_{4}}\left(\hat{f_{3}}\left(\hat{f_{2}}\left(\hat{f_{1}}\left(x\right)\right)\right)\right)=W_{4}W_{3}W_{2}W_{1}x=\frac{1}{2}\left|\right|W_{4}W_{3}W_{2}W_{1}x-y{\left|\right|}_{2}^{2}$$

Hence, the gradients are updates or weights with the backpropagation algorithm derived with respect to the input or the parameters of the respective layer using non-linear functions, for instance, the Rectified Linear Unit ReLU(x).

#### 2.3.1. CNN-Based Classification

Let us assume that a CNN architecture used for binary classification delivers its output $\hat{y}$ from an input ***x*** convolved with a learned kernel, defining the model $\phi :X⟶R^{D}$ parametrized by ***θ***, to produce a feature map ***a***. Then, the feature map ***a*** becomes input to a classifier $f:R^{D}⟶R$ parametrized by ***w*** and passing through a non-linear operator, such as the sigmoid function σ. Formally, we assume an input ***x***, that is

(4) a=ϕ(x;θ)

followed by

(5) z=f(a;w)

and then

(6) $$\hat{y}=\sigma \left(z\right)=\frac{1}{1+e^{z}}$$

In the case of supervised learning, the learning task relies on a set T with *n* data points, each one provided with both the input sample and its ground truth label, that is:

(7) $$T={\left\{x_{i},y_{i}\right\}}_{i=1}^{n}$$

Then, each of the model’s outputs $\hat{y}$ is arithmetically compared to the ground truth label *y* to compute the loss of the learning iteration using a function $J\left(y,\hat{y}\right)=\sum_{i=0}^{m}\left|y^{\left(i\right)}-{\hat{y}}^{\left(i\right)}\right|$.

#### 2.3.2. Transfer Learning

In many scenarios, the amount of data necessary for effective learning via neural networks is prohibitive. In these cases, one must resort to prior learning (training) obtained by means of a sufficiently similar dataset; for example, a deep network trained for general object recognition can be tuned for the classification of mechanical parts in an assembly line. This sort of prior learning is known as transfer learning, according to which the model is first trained on a similar task with abundant data before learning the target task. In the case of tasks involving images, the majority of the layers are dedicated to extracting descriptive features from the input, while the last layers use these features as input for classification.

When using transfer learning, the layers dedicated to feature extraction are kept from one task to the other, with only the last layers tuned for a different task. Recently, it was shown that a careful optimization strategy for transfer learning not only significantly boosted the training procedure but also, in many cases, improved the generalized performance. In this paper, we compare a model that is built on lightweight and dense CNNs with models that are trained based on prevailing fine-tuning methods.

Transfer learning covers a broad range of learning tasks, such as meta-learning [41], continual-learning [42], and task domain-adaptation [43]. Typically, a transfer learning strategy consists of a pre-trained network built over a different task and such model is adapted with fine-tuning over all deep layers, or a partial number of layers, for a new classification task with a different domain. Formally, let $X=\{x_{1},\ldots ,x_{n}\}$ be source data sampled from the source domain distribution DS. Consider also $Y=\{y_{1},\ldots ,y_{m}\}$ as the target data from the target domain distribution DT. Classical machine learning assumes similar distributions, i.e. DS∼DT, while transfer learning aims to solve the divergence between domain distributions.

## 3. Experimental Analysis

### 3.1. DAIS Image Dataset

We colleted 210 images using DAIS; our tests were performed by the Structures, Materials and Propulsion Laboratory of the IAR/NRCC and Diffracto Limited, under a collaborative agreement. To determine the effect that skin thickness loss has on the stress in a joint, two conditions were studied: (1) no corrosion, (2) corrosion caused by pillowing with effective skin thickness reduction and expansion.

**Figure 4:**
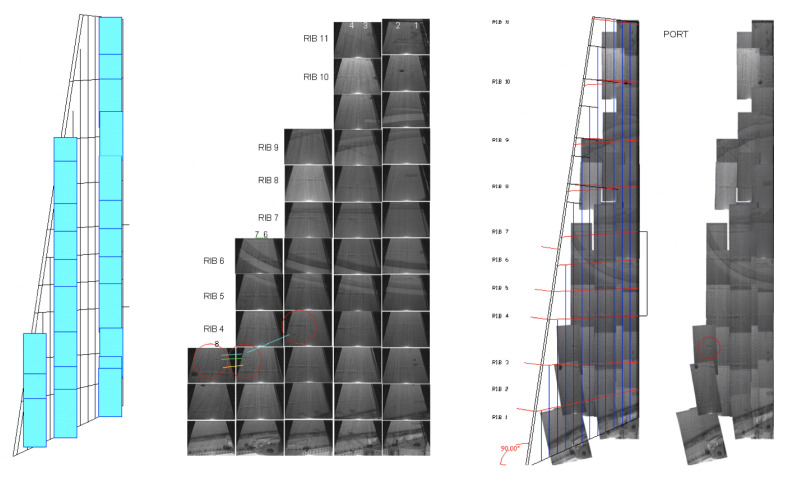
Wing inspection images created with DAIS 250C. Images are composed as a mosaic to cover the entire wing. Each image sample is enumerated in order to control the inspection. A specialist then perform the inspection of each sample.

During the image acquisition from aircraft-class Boeing 727 and AirBus 300, we noticed that the volumetric increase associated with the corrosion products accelerated the volume of the parent material lost, which is much higher than was initially thought. This volumetric increase raised concerns regarding the possible effects of pillowing on the structural integrity of corroded fuselage joints. A total of 200 fuselage joint images (TIFF-type) with size 640×480 were selected for inclusion in the dataset. There was no overlap between any two images. All images were inspected by a team of specialists to validate whether the images were de facto corroded or not. Figure 3 shows samples with and without corrosion, as described in this study. Figure 4 illustrates how image samples were collected for a wing inspection by using DAIS sensor.

### 3.2. Experimental Setup and Training

In this section, we describe the experimental setup for testing and training the CNN-based architectures. For validating the models, we used five-fold cross-validation. Before training, we proceeded with cropping and resizing. We selected squared crops starting at the center of the images and then peripherally, but with no overlapping; then, we resized the images according to the input of each CNN architecture. The images for training and testing were from different regions of the aircraft. Our idea is to avoid similarity between images from the test set and the training set to achieve an ideal generalized model.

The model parameters were initialized with weights from the same architecture pre-trained on the Imagenet competition [29] to reduce the training time. At present, there exist several image classification networks that provide a good trade-off between accuracy, robustness, and speed, such as Inceptionv3 [40], ResNet-101 [44], NasNet [45], SqueezeNet [38], and two variants of DenseNet [39], DenseNet-121 and DenseNet-201. Furthermore, several studies are focusing on improving these networks on specific image classification tasks. In this work, we used different architectures, including DenseNet and SqueezeNet, since it these are most accurate detection deep neural nets according to the this study [46]. Additionally, in image recognition, classic ResNet-like architectures are still considered state-of-the-art [47,48].

Models were trained and tested on a desktop computer with Intel Xeon W-10885M Processor, 16 M Cache, 5.30 GHz, 64 GB memory, and NVIDIA Titan RTX Graphics Card 24 GB running on the Ubuntu 18.04 operating system. For binary classification, we computed the final probabilities using normalization softmax with loss function cross-entropy. A learning rate of 0.001 was used, while the number of iterations was set to 75 epochs using batches of four images, and stochastic gradient descent optimization. For regularization and convergence, we employ classic L2 normalization with a ratio of 0.0005. Data augmentation methods include several types of transformation, such as shearing with angle in $\left[-15^{\circ },15^{\circ }\right]$ and with scale factor [1/1.6,1.6]. The results were not compared with previous works in corrosion, since our data collection was based on a different sensor. We compared our methodologies over two neural network settings using botha lightweight and a dense network.

### 3.3. Evaluation Metrics

To evaluate the DL-based methodology, we used common classification metrics, which were the precision, recall, f-measure, and accuracy:

(8) $$Precision=\frac{TP}{TP+FP}\;\;Recall=\frac{TP}{TP+FN}$$

(9) $$Accuracy=\frac{TP+TN}{TP+FN+FP+TN}\;\;F-measure=\frac{2\times Precision\times Recall}{Precision+Recall}$$

where TP, FP, FN, and TN correspond to true positives, false positives, false negatives, and true negatives, respectively.

## 4. Results

In our experiments, we first evaluated the performance of the CNN models in classifying the images. Next, we analyzed the behavior in building the models using learning curves; error bars for each epoch were plotted. We also demonstrated the effectiveness of the architectures comparing the performance with different metrics. Then false positives were displayed to visually discriminate the misclassified images.

### 4.1. Performance Evaluation

**Figure 5:**
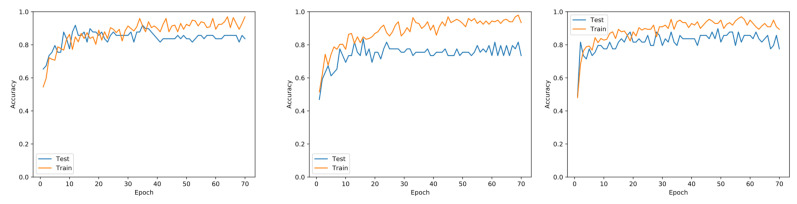

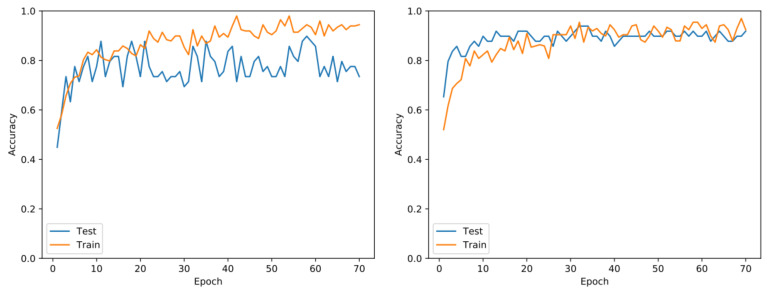
Learning curves for training and testing using the DenseNet architectecture, whose accuracy achieved the top results. Each plot corresponds to a five-fold split.

**Figure 6:**
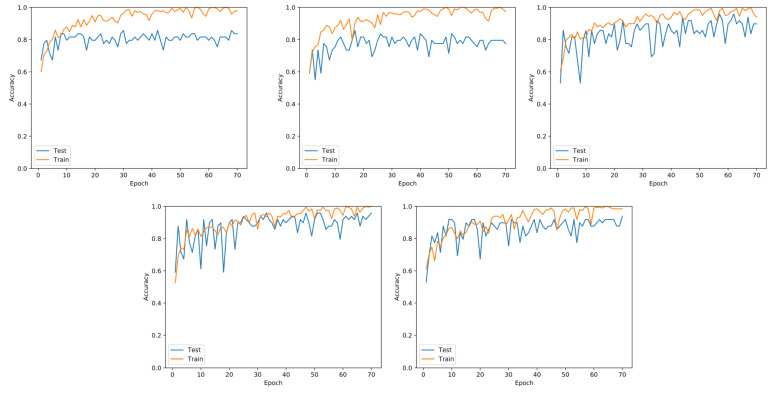
Learning curves for training and testing using the SqueezeNet architectecture. Although it is considered a light weight architecture, SqueezeNet achieved the second best results. Each plot corresponds to a five-fold split.

The training of the methods was performed with the parameters described in Section 3.2. The loss curves of two models—DenseNet and SqueezeNet—are presented in Figure 5 and Figure 6, respectively. These learning curves indicated no evidence of overfitting because the accuracy values for training and validation were similar, i.e., although there were some peaks during testing, they did not cross the training curve after 20 epochs in each fold of the cross-validation.

We also experimented with variants of the same architectures for comparison. The state-of-the-art DenseNet with two variations in terms of the number of layers was used. Our dataset did not consider a class imbalance since the number of samples did not diverge for positive and negative samples. It was quite important to point that we did not obtain the peaks of accuracy that we showed in Table 1. Instead, the number of max epochs was set to 75; this was due to the fast convergence of the models, whose learning curves indicated no variability, and the results demonstrated high accuracy for image classification. We tested with a larger number of epochs; however, the results did not change significantly.

Similarly, we tested the same idea using SqueezeNet [38], a lightweight convolutional neural network. Figure 6 shows the learning curves for all five folds. The behavior of the curves was very similar to the DenseNet CNN, which implies that the lightweight network is suitable for this case.

We did not compare the performance of our study with other existing corrosion datasets reported in the literature, since the images yielded with the DAIS sensor had never been automatically evaluated during Deep Learning-based image classification. Nevertheless, we conductd the experiments with Inceptionv3, ResNet-101, NasNet, SqueezeNet, and two variants of DenseNet, DenseNet-121 and DenseNet-201. The results are given in Table 1 with remarkably high scores. Particularly, the accuracy for the DenseNet architecture was the highest with 92.2%, a maximum value considering all the epochs, followed by SqueezeNet (91.6%) and Inceptionv3 (90.6%). We also showe the results for the maximum and average concerning the last 15 epochs, as well as for the last five epochs. NasNet and Resnet-101 achieved the worst results, with (88.1%) and (88.4%), respectively.

**Figure 7:**
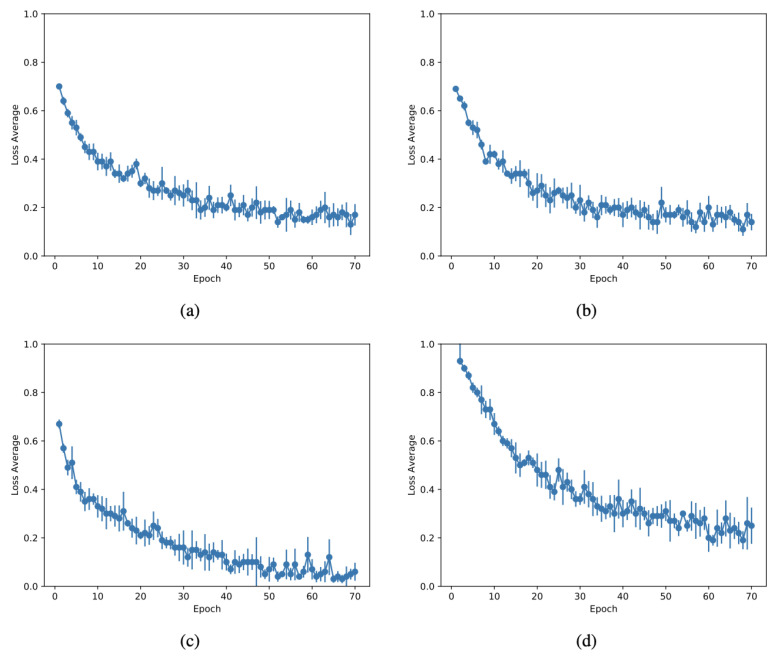
Loss curves with error bars depicting the loss for the epochs for the different architectures evaluated: DenseNet (**a**), ResNet (**b**), SqueezeNet (**c**), and InceptionV3 (**d**).

Figure 7a through Figure 7d present the loss curves demonstrating the fast pace of convergence after just a few epochs. It is possible to conclude that such a dataset is well-suited for transfer learning, benefiting from different image-related problems used in the original trainings.

We compared the architecture using the precision, recall, accuracy, and F-measure, as presented in Table 2. The accuracy is the same maxixum value from Table 1. The value of precision for DenseNet [39] indicates a high test accuracy and a robust classifier with 93.2%, compared to Xception [49] with 89.2%— five-fold dataset splitting. Furthermore, although DenseNet relies on a large number of layers when compared with the other architectures, it provides the best results considering all the metrics, including Recall and F-measure. The large values indicate the feasibility of adopting a lightweight network, such as NasNet; however, the dense architectures still perform better as shown in Table 2. A complete comparison in the performance of different Deep Learning models is outlined in the same table.

### 4.2. Discussion

**Figure 8:**
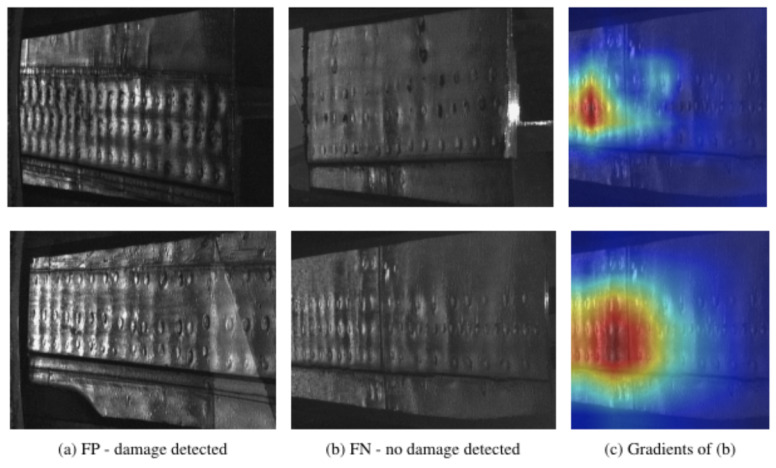
Examples of images that the Deep Learning architecture DenseNet-201 did not predict well. Column (**a**) corresponds to images misclassified with corrosion or false positives (FP), while column (**b**) includes images with misclassified corrosion detected or false negatives (FN). In (**c**), we use Grad-CAM for the visual interpretability of the DenseNet-201’s gradients over the image in (**b**).

Deep learning has the potential to revolutionize the way companies perform aviation maintenance. Here, we investigated the performance of a deep neural network, and found the best performance with DenseNet with 201 layers through visually comparing the misclassification, that is, the false positives and false negatives. Figure 8a presents cases where the model predicted false positives for corrosion, and Figure 8b presents false negatives with non-corrosion.

In Figure 8c, we show the visual attention spot of the model of samples in b. The samples were selected when the number of epochs achieved the maximum accuracy. Although we evaluated our model using different Boeing 727-class aircrafts, it is important to note that throughout a unique fuselage, the joints change in terms of the skin (sheet metal) thickness, number of layers, and rivet types. Therefore, for robustness, our training set included a diverse set of lap joints from different aircraft considering specific locations of the fuselage.

Based on our findings, we can consider that the models built are effective for a large variety of aircraft parts, once images were taken with different angles, variation in distance between the device and the inner fuselage, and distinct fuselage aircraft, e.g., Boeing 727 and AirBus 300. Poor resolution images might affect the results negatively, an effect observed in several AI and computer vision systems fed with unseen and outlier samples. This could be mitigated by adding low-resolution samples in the training phase to make the models even more robust to new input tests. However, cutting-edge DAIS devices capture images with high resolution pixels, including the version used in this study.

#### Visual Interpretability

Afterward, we interpreted the functioning of our network using the technique GradCam [50] for correctly classified samples. Grad-CAM essentially uses the gradient information with saliency maps via the backpropagation of class scores with respect to the pixel intensities of the input image. Such mapping generates visual explanations from CNN-based models and is illustrated by means of a heatmap, where parts of a given image were the most decisive for the classification.

**Figure 9:**
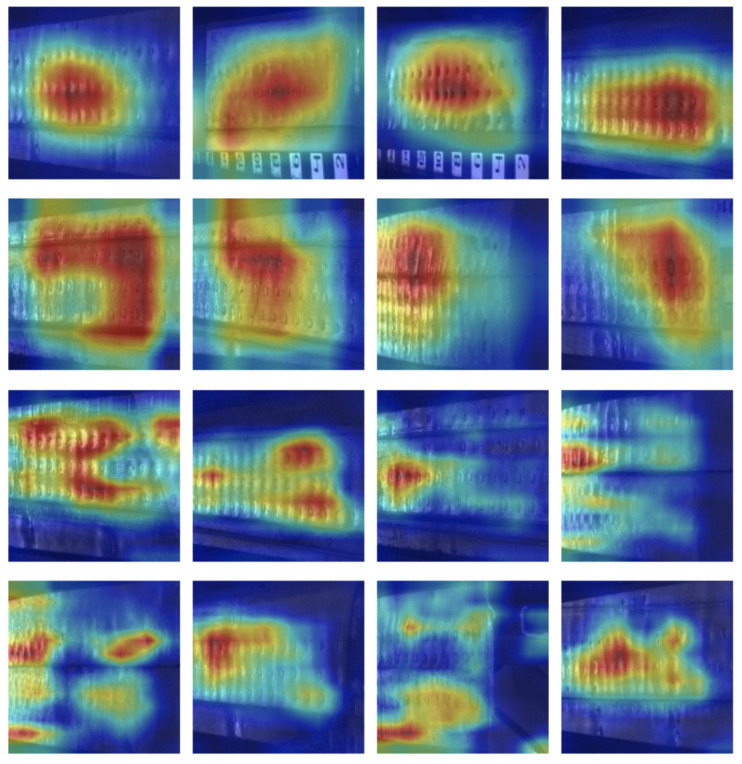
Visualization results through class-activated maps overlaid on input true positive images together with the raw images. The maps were extracted from the last convolution layer using the algorithm GradCam for DenseNet-201 (first two rows) and SqueezeNet (last two rows). The heat maps stand for the larger heights learned during the training.

Here, we provide two sets of eight examples in Figure 9, the first two rows correspond to the deep model DenseNet-201 and, the latter, stand for the lightweight model SqueezeNet. The network learned to identify the regions of highest-stress, that is, where the planarity of the fuselage appeared not to be smooth in comparison to its neighbors.

We note that Boeing and Airbus aircraft construction is reasonably similar across their aircraft families, such as B727, B737, and B7- and A300, A310, A320, and A330. The Douglas and Lockheed aircraft is slightly different; nevertheless, with well-trained models, we presume the potential to achieve good results by extending our training dataset with D-Sight images from such class of aircraft.

As a last remark, we reaffirm that our results are encouraging; the quality of the classifications considering the best architecture even rivaled the human ground-truth judgment, which also might fail since some images are very similar in spectrum, conveying patches of corrosion in rivets and in some joint parts. In this paper, we took the first step for automatic corrosion recognition through a data-driven approach. Thereby, the ability to successfully classify corrosion on a human level with images opens an exciting new direction based on cutting-edge Artificial Intelligence.

## 5. Conclusions and Future Works

This paper presented a CNN approach to detect corrosion in aircraft fuselage through images produced by the D-Sight Aircraft Inspection System (DAIS). The manual visual inspection of aircraft structural integrity has a significant chance of inadequately identifying corrosion, and there is extensive time and human labor involved. For computer-aided aircraft inspection, we evaluated multiple neural network parametrizations and found that transferring features from pre-trained CNNs was an effective solution for corrosion detection. In an automated manner, our results archived an accuracy of 90.2% with InceptionV3 and 92.2% with DenseNet. These results demonstrated promising performance in improving the state of the art in aircraft corrosion detection.

We notice that, due to the expensive image acquisition and manual labeling, we were forced to work with a relatively small amount of labeled data. Hence, it is expected that our methodology could use more labeled data to improve its generalization power with respect to other aircraft manufacturers beyond Boeing and Airbus. Future work might include different approaches of task adaptation to improve and extend the model’s performance over different aircraft structures and measure the impact of skin thickness—for instance, distinct Boeing-class fuselages and military helicopters might present thicker materials. In the face of the success of our computational model, we are considering deploying a DNN-based model trained with Boeing and AirBus aircrafts on corrosion detection tests of military jets and fighter helicopters.

## Figures and Tables

**Table 1:** Results regarding the learning curves, including the max value for all epochs, average for the last 15 epochs, max value for the last 15 epochs, and average for the last 5 epochs.

			Accuracy		
Architecture	Reference	Max Value	Avg Last 15	Max Last 15	Avg Last 5
Inceptionv3	[40]	90.6±0.02	84.8±0.02	88.9±0.02	84.7±0.02
Resnet-101	[44]	89.1±0.03	81.3±0.02	86.9±0.04	81.8±0.03
NasNet	[45]	88.1±0.05	67.1±0.05	70.6±0.05	68.3±0.05
SqueezeNet	[38]	91.6±0.04	86.2±0.05	90.6±0.05	86.6±0.04
DenseNet-121	[39]	87.6±0.03	82.9±0.04	87.3±0.03	81.7±0.04
DenseNet-201	[39]	92.2±0.02	83.6±0.03	88.6±0.04	84.4±0.03

**Table 2:** Comparison of the main architectures in the literature.

Architecture	Reference	Precision	Recall	Accuracy	F-Measure	Size (MB)	Depth
Inceptionv3	[40]	89.2±0.05	84.7±0.04	90.6±0.02	86.8±0.05	92	126
Resnet-101	[44]	90.0±0.06	88.2±0.03	89.1±0.03	89.7±0.04	171	101
NasNet	[45]	88.1±0.05	93.5±0.08	88.1±0.05	89.2±0.07	23	-
SqueezeNet	[38]	89.2±0.04	88.4±0.04	91.6±0.04	85.4±0.02	0.5	18
DenseNet-121	[39]	91.3±0.08	84.2±0.04	87.6±0.03	89.7±0.03	33	121
DenseNet-201	[39]	93.2±0.05	93.3±0.04	92.2±0.02	91.4±0.05	80	201

## Data Availability

Data is entirely commercial property and it will be not available. Additionally, extra data has been collected to expand the experiments with AI learning models before turning it public.

## Sample Availability

The datasets generated during and/or analyzed during the current study are not publicly available due to a Non-Disclosure Agreement, but are available from the corresponding author on reasonable request.
